# Morphological Neuroimaging Biomarkers for Tinnitus: Evidence Obtained by Applying Machine Learning

**DOI:** 10.1155/2019/1712342

**Published:** 2019-12-13

**Authors:** Yawen Liu, Haijun Niu, Jianming Zhu, Pengfei Zhao, Hongxia Yin, Heyu Ding, Shusheng Gong, Zhenghan Yang, Han Lv, Zhenchang Wang

**Affiliations:** ^1^School of Biological Science and Medical Engineering, Beihang University, Beijing, China; ^2^Department of Radiation Oncology, University of North Carolina Healthcare, North Carolina, USA; ^3^Department of Radiology, Beijing Friendship Hospital, Capital Medical University, Beijing, China; ^4^Department of Otolaryngology Head and Neck Surgery, Beijing Friendship Hospital, Capital Medical University, Beijing, China

## Abstract

According to previous studies, many neuroanatomical alterations have been detected in patients with tinnitus. However, the results of these studies have been inconsistent. The objective of this study was to explore the cortical/subcortical morphological neuroimaging biomarkers that may characterize idiopathic tinnitus using machine learning methods. Forty-six patients with idiopathic tinnitus and fifty-six healthy subjects were included in this study. For each subject, the gray matter volume of 61 brain regions was extracted as an original feature pool. From this feature pool, a hybrid feature selection algorithm combining the *F*-score and sequential forward floating selection (SFFS) methods was performed to select features. Then, the selected features were used to train a support vector machine (SVM) model. The area under the curve (AUC) and accuracy were used to assess the performance of the classification model. As a result, a combination of 13 cortical/subcortical brain regions was found to have the highest classification accuracy for effectively differentiating patients with tinnitus from healthy subjects. These brain regions include the bilateral hypothalamus, right insula, bilateral superior temporal gyrus, left rostral middle frontal gyrus, bilateral inferior temporal gyrus, right inferior parietal lobule, right transverse temporal gyrus, right middle temporal gyrus, right cingulate gyrus, and left superior frontal gyrus. The accuracy in the training and test datasets was 80.49% and 80.00%, respectively, and the AUC was 0.8586. To the best of our knowledge, this is the first study to elucidate brain morphological changes in patients with tinnitus by applying an SVM classifier. This study provides validated cortical/subcortical morphological neuroimaging biomarkers to differentiate patients with tinnitus from healthy subjects and contributes to the understanding of neuroanatomical alterations in patients with tinnitus.

## 1. Introduction

Tinnitus, the perception of sounds in the absence of any external sound stimuli, is experienced by 15% of the global population. Tinnitus presents as a variety of sounds, and it is typically sensed as ringing, hissing, or buzzing, among other sounds, in the ears or the head [1, 2]. For most patients, the etiology of tinnitus is not quite clear, and this type of tinnitus is usually defined as idiopathic tinnitus in the clinic. Patients with tinnitus often suffer from hearing loss, stress, and sleep disturbance [3]. Since there are no effective treatments for tinnitus, it is important to understand the sensory and cognitive mechanisms that may directly or indirectly be associated with alterations in the cortical/subcortical architecture [4].

With the use of advanced neuroimaging techniques, previous studies have suggested that patients with tinnitus may exhibit anatomical alterations in auditory- and non-auditory-related brain areas, as detected by voxel-based morphometry (VBM) analysis [5–9]. Brain morphological changes in auditory-associated brain areas, including the primary and secondary auditory cortex (PAC/SAC) located in the temporal gyrus, as well as in non-auditory-related brain areas (especially the limbic system), have been commonly reported in previous studies [10, 11]. Several inherent networks—including but not limited to the default mode network (DMN), dorsal attention network (DAN), and frontal-parietal network—have also been implicated in tinnitus [12, 13]. Brain morphology studies in tinnitus have generally been widespread, and the results obtained by different studies show only partial agreement. It is quite difficult to reconcile previous results due to their inconsistency and heterogeneity. The inconsistency may be related to different groups of enrolled patients, small sample sizes, and differences among patients in terms of the kind of perceived sound, degree of distress, disease duration, presence of hyperacusis, and hearing loss status. The key cortical/subcortical morphological neuroimaging biomarkers that characterize tinnitus remain unclear.

Morphological neuroimaging biomarkers may not be best explored in only one research study. Rather, it would be better to combine the results with those of previous studies, comprehensively summarize various published results, and then extract the key features of tinnitus patients. Machine learning, an artificial intelligence methodology concerned with the implementation of computer software that learns autonomously, is a promising approach for extracting features from large information sources [14]. Specifically, the support vector machine (SVM) is a supervised learning model with associated learning algorithms that maximize the distance of a hyperplane for classification and regression analysis. Both linear and nonlinear data can be processed by the SVM method with superior generalization performance [15]. It has been successfully applied to explore morphological neuroimaging biomarkers for the classification and diagnosis of different subsets of neurological diseases, including Alzheimer's disease (AD) and schizophrenia [16, 17]. Based on published morphological studies of patients with tinnitus, the SVM method could also effectively extract neuroimaging biomarkers for tinnitus.

In this study, we hypothesized that there may be several cortical/subcortical morphological neuroimaging biomarkers that can characterize tinnitus. To test our hypothesis, we first summarized brain regions with significant morphological alterations reported in previous studies and extracted the gray matter (GM) volume of these brain regions as an original feature pool. Then, a stable and efficient classifier was generated to analyze the summarized brain areas, followed by fivefold cross-validation to evaluate the accuracy of the classifier in forty-six tinnitus patients and fifty-six healthy controls based on the SVM model. The brain regions that may effectively differentiate patients from healthy subjects were then extracted as the key cortical/subcortical morphological neuroimaging biomarkers. Our study provides validated evidence of neuroanatomical biomarkers for differentiating patients with tinnitus from healthy subjects.

## 2. Materials and Methods

### 2.1. Subjects

This study was approved by the medical research ethics committees and institutional review board. Written informed consent was obtained from each subject.

For feature selection and model training, forty-six patients with tinnitus were recruited in this study. All of the subjects were recruited from Beijing Friendship Hospital. The inclusion criteria were as follows: (1) patients without significant hearing loss (the subjects had hearing thresholds less than 25 dB HL at frequencies of 0.250, 0.500, 1, 2, 3, 4, 6, and 8 kHz determined by pure-tone audiometry (PTA) examination) and (2) patients with a symptom duration longer than 3 months. The exclusion criteria were as follows: (1) patients diagnosed with pulsatile tinnitus; (2) patients with hyperacusis; (3) patients with neurological disease, such as dementia or AD; (4) patients with any kind of otological condition, such as Meniere's disease or otosclerosis; and (5) patients contraindicated for magnetic resonance imaging (MRI) examination. The Tinnitus Handicap Inventory (THI) score was also acquired in the patient group to assess the severity of tinnitus and tinnitus-related distress. According to the score, tinnitus was divided into five levels: mild (1-16), light (18-36), moderate (38-56), severe (58-76), and catastrophic (78-100) [18]. Fifty-six age- and sex-matched healthy controls were also enrolled as healthy subjects. The exclusion criteria for the healthy controls were the same as those listed above. The characteristics of the subjects are presented in Table 1.

### 2.2. MRI

Images were acquired using a 3.0T GE Signa Excite MR scanner (General Electric Medical Systems, Milwaukee, WI, USA) equipped with an eight-channel, phased-array head coil. Parallel imaging was employed in data acquisition. High-resolution 3D structural images were acquired using a 3D-BRAVO pulse sequence with the following acquisition parameters: TR (repetition time) = 8.5 ms; TE (echo time) = 3.3 ms; TI (inversion time) = 450 ms; matrix = 256 × 256; field of view (FOV) = 24 cm × 24 cm; and slice thickness = 1 mm without gap. In total, 196 slices were obtained from each subject.

### 2.3. Image Processing

Image preprocessing was performed with the VBM8 toolbox in the SPM8 software package (Statistical Parametric Mapping, Wellcome Department of Cognitive Neurology, London, UK) running in MATLAB (MathWorks, Natick, MA, USA). The procedures for image preprocessing have been described in detail [19]. Briefly, image processing in this work included spatial normalization using the Montreal Neurological Institute (MNI) 152 template and segmentation of the GM, white matter (WM), and cerebrospinal fluid (CSF). Only the GM images were analyzed in this study.

In this study, several morphologically relevant papers published with five years before the start of the study were summarized, and the results of the papers were collated [4, 5, 8, 20, 21]. Based on the purpose and method of this study, the methods used in previous studies were not limited. Finally, sixty-one cortical/subcortical brain regions were summarized as the targeted structures for analyzing the anatomical changes in tinnitus patients (listed in Table 2). These brain regions roughly cover the findings of existing studies. Brain regions reported to be associated with hearing loss were not included in this study. The peak intensity of each brain region was labeled in MNI space. For each brain region, the region of interest (ROI) was defined as a sphere with a radius of 5 mm with its peak MNI coordinates as the center using the MarsBaR toolbox [22]. The ROI volumes were measured and recorded as the original features of each patient for classification.

### 2.4. Feature Selection Algorithm

**Figure 1:**
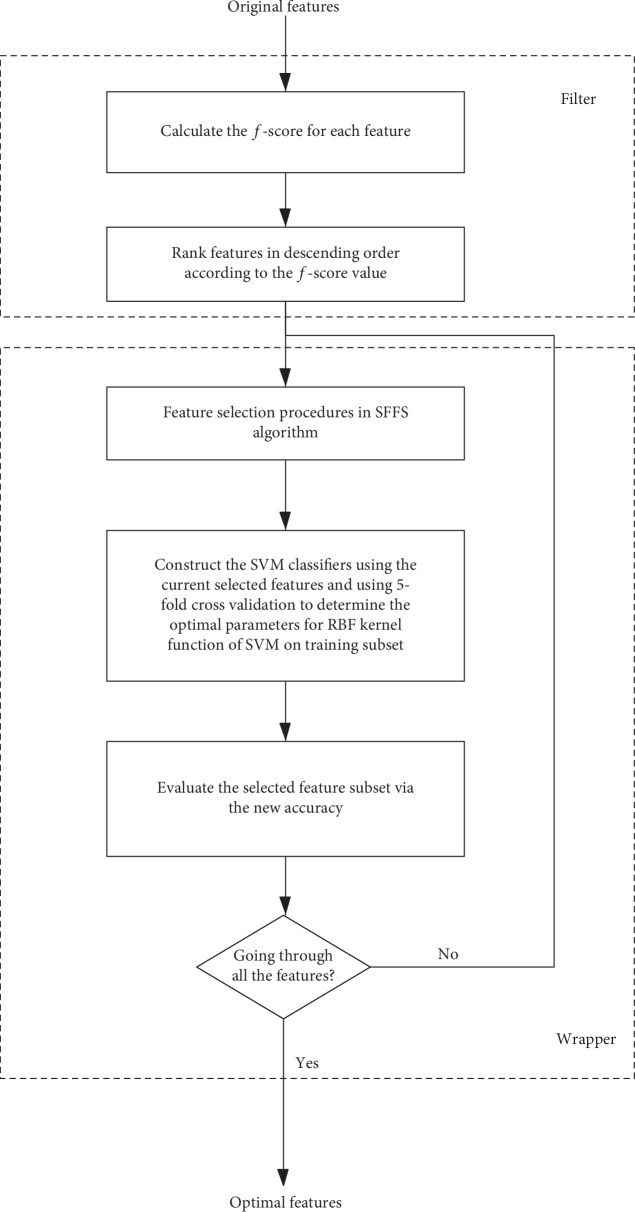
Hybrid feature selection algorithms.

Feature selection plays an important role in the classification process. Feature selection algorithms are mainly divided into two categories: the filter and wrapper methods [23]. The filter method is independent of the classifier and allows rapid training. The wrapper method requires a long training time since it depends on the classifier, and the performance of the selected feature subsets is evaluated by the accuracy of the classifier. However, the classification performance of the wrapper method is superior to that of the filter method. A hybrid feature selection algorithm containing both types of methods was used in this study. In general, stable and efficient classifiers were generated by the following steps [24]. First, the filter method was adopted to rank the features according to the *F*-score, as described below. Next, sequential forward floating selection (SFFS) was used as the wrapper method to select features according to the accuracy of the SVM classifier. Finally, the features that optimized the performance of the SVM classifier were obtained. Fivefold cross-validation was used in the current study.  Figure 1 illustrates the main procedures of the hybrid feature selection algorithm.

The *F*-score is a criterion used to rank the importance of a feature between different sets of real numbers [25]. The *F*-score was used to rank the features according to two sets of feature values in this study. Given the training vector *x*_*i*_ ∈ *R*^*m*^(*k* = 1, 2, ⋯, *n*), the sample size of the positive and negative subset was *n*_+_ and *n*_−_, respectively. The *F*-score of the *i*^th^ feature, *F*_*i*_, was calculated as follows:

(1) $$\begin{array}{c} F_{i}=\frac{{\left({{\bar{x}}_{i}}^{\left(+\right)}-{\bar{x}}_{i}\right)}^{2}+{\left({{\bar{x}}_{i}}^{\left(-\right)}-{\bar{x}}_{i}\right)}^{2}}{\left(1/n_{+}-1\right)\sum_{k=1}^{n_{+}}{\left({x_{k,i}}^{\left(+\right)}-{{\bar{x}}_{i}}^{\left(+\right)}\right)}^{2}+\left(1/n_{-}-1\right)\sum_{k=1}^{n_{-}}{\left({x_{k,i}}^{\left(-\right)}-{{\bar{x}}_{i}}^{\left(-\right)}\right)}^{2}}, \end{array}$$

where $\bar{x}$, ${{\bar{x}}_{i}}^{\left(+\right)}$, and ${{\bar{x}}_{i}}^{\left(-\right)}$ are the average value of the *i*^th^ feature in the whole dataset, in the positive subset, and in the negative subset, respectively, and *x*_*k*,*i*_^(+)^ and *x*_*k*,*i*_^(−)^ are the *i*^th^feature of the *k*^th^ instance in the positive and negative subsets, respectively. The larger the *F*_*i*_, the more discriminative the *i*^th^ feature.

After determining the *F*-score, the features were ranked in descending order according to their *F*_*i*_ value. The SFFS feature selection strategy was then used, as previously proposed by Pudil et al. [26]. The features were added in feature sets in sequence, and feature retention was based on the accuracy of the SVM classifier at each step. If the accuracy of the SVM classifier with a new feature set did not increase, the new feature was removed from the feature set.

The SVM method is a machine learning technique initially proposed by Vapnik in the 1990s [27]. The basic idea of the SVM method is to obtain the largest-margin classifier using a kernel function. To determine the optimal SVM classifier, the radial basis function (RBF) kernel, defined as *K*(*x*_*i*_, *x*_*j*_) = exp(*γ*|*x*_*i*_ − *x*_*j*_|^2^), was adopted here [28]. The grid search algorithm with 5-fold cross-validation was used to search for the best parameter pairs (*C*, *γ*) for the RBF kernel. The search range for *C* and *γ* was log_2_*C* = {−5, −4, ⋯, 4, 5} and log_2_*γ* = {−5, −4, ⋯, 4, 5}, respectively.

Feature selection was performed with MATLAB code written in-house. The pseudocode of the feature selection procedure is described here:

**Step 1 .**

Group subjects: the tinnitus patients were divided into five groups, consisting of 10, 9, 9, 9, and 9 patients. Similarly, the 56 healthy subjects were divided into five groups, consisting of 12, 11, 11, 11, and 11 subjects. Then, the patients and healthy subjects were combined together into groups of 22, 20, 20, 20, and 20, respectively. During the feature selection and training process, four groups were selected as the training set at each step, and the remaining group was selected as the test set.

**Step 2 .**

Calculate the *F*-score: for each training set, the *F*-score was computed for each feature using equation (1), and the features were ranked in descending order according to the *F*-score.

**Step 3 .**

Build a classifier: each training set was randomly divided into five groups using a 5-fold cross-validation method. Each time, four groups were selected as the training subset, and the remaining group was used as the test subset. For each training subset, the sorted features were added to the feature set in turn; the feature set was initially empty. The SVM classifier was constructed using the selected features, and the optimal parameters (*C*, *γ*) of the SVM classifier were determined using the grid search algorithm.

**Step 4 .**

Apply search strategy: according to the SFFS strategy and the accuracy of the classifier, if the new accuracy was not improved, the newly added feature was removed from the feature subset. Otherwise, the feature was retained.

**Step 5 .**

Steps 3 and 4 were repeated until all features were selected. The accuracy of the test set was calculated.

### 2.5. Statistical Analysis

To obtain a generalized SVM classification model, it was necessary to select the appropriate *C* and *γ*; thus, the grid search and cross-validation methods were adopted. The average classification accuracy in the training set for each set of *C* and *γ* was calculated, and the set of *C* and *γ* with the best classification accuracy in the training set was selected as the optimal group of parameters for the SVM model. Then, the corresponding test set was used for performance testing, and the classification accuracy was calculated. The feature (brain region) combination with the best classification performance effectively differentiated tinnitus patients from healthy subjects.

Additionally, the performance of the SVM classifier was evaluated by creating the receiver operating characteristic curve (ROC) and calculating the area under the curve (AUC). Additionally, Pearson's correlation analyses for evaluating the THI score and the volume of brain regions that could effectively differentiate tinnitus patients from healthy controls were conducted using SPSS software (version 20.0; SPSS, Chicago, IL). *p* < 0.05 was considered statistically significant.

## 3. Results

The highest accuracy and corresponding parameters (*C*, *γ*) were obtained. After the grid search during the feature selection procedure, the optimal parameters (*C*, *γ*) of the SVM classifier were adjusted as follows: *C* was set to 2, and gamma was set to 8.

In all, 13 features were selected from the 61 original features.  Table 3 shows that the accuracy of the training set and the test set was 80.49% and 80.00%, respectively.

**Figure 2:**
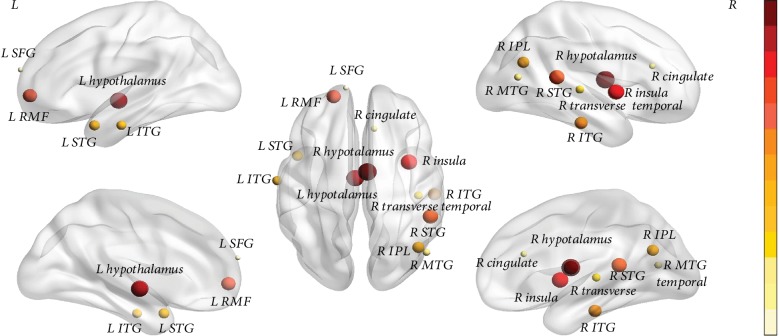
Brain regions that could effectively differentiate tinnitus patients from healthy controls with the highest accuracy. The color bar indicates the degree of importance of brain region for classification. The hotter the color and the larger the ball, the more significant the brain region is for classification. R = right hemisphere; L = left hemisphere; STG = superior temporal gyrus; SFG = superior frontal gyrus; RMF = rostral middle frontal gyrus; IPL = inferior parietal lobule; ITG = inferior temporal gyrus; MTG = middle temporal gyrus; SFG = superior frontal gyrus.

As shown in Figure 2 and Table 4, after controlling for the effect of aging, the combined features with the highest classification accuracy revealed the brain regions that could effectively differentiate tinnitus patients from healthy controls. Those brain regions included the bilateral hypothalamus, right insula, bilateral superior temporal gyrus (STG), left rostral middle frontal gyrus, bilateral inferior temporal gyrus (ITG), right inferior parietal lobule (IPL), right transverse temporal gyrus, right middle temporal gyrus (MTG), right cingulate gyrus, and left superior frontal gyrus (SFG).

**Figure 3:**
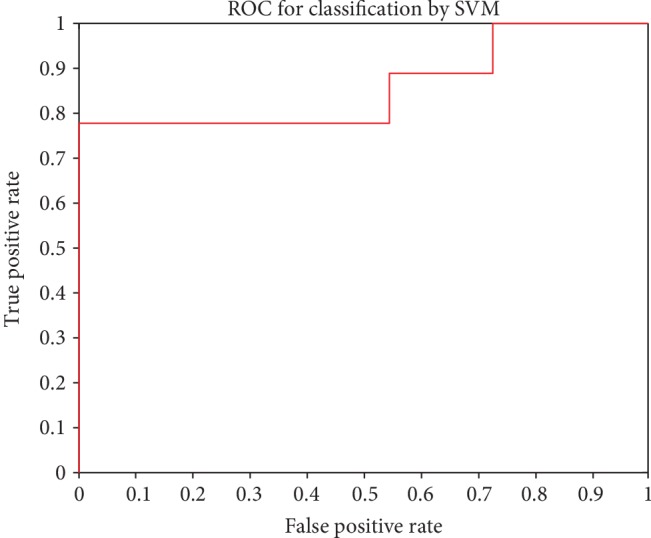
ROC (receiver operating characteristic) curve for SVM classification.

The AUC was 0.8586 for the hybrid feature selection algorithm.  Figure 3 shows the ROC curve for the set of 13 brain regions (shown in Table 4) and the probability scores for all 102 data points in our dataset.

**Figure 4:**
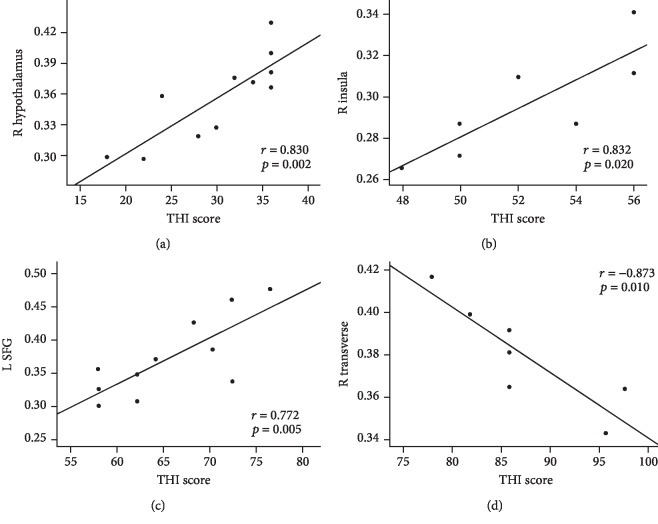
Correlations between THI score and volume of brain regions. (a) Correlations between THI score and volume of the right hypothalamus in light tinnitus (*r* = 0.830, *p* = 0.002). (b) Correlations between THI score and volume of the right insula in moderate tinnitus (*r* = 0.832, *p* = 0.020). (c) Correlations between THI score and volume of the left SFG in severe tinnitus (*r* = 0.772, *p* = 0.005). (d) Correlations between THI score and volume of the right transverse in catastrophic tinnitus (*r* = −0.873, *p* = 0.010). R = right hemisphere; L = left hemisphere; SFG = superior frontal gyrus.

Pearson's correlation analyses revealed that THI score was positively correlated with the volume of the right hypothalamus (*r* = 0.830, *p* = 0.002), right insula (*r* = 0.832, *p* = 0.020), and left SFG (*r* = 0.772, *p* = 0.005) in light, moderate, and severe tinnitus, respectively. Additionally, the THI score was negatively correlated with the volume of the right transverse gyrus in catastrophic tinnitus (*r* = −0.873, *p* = 0.010) (Figure 4).

## 4. Discussion

Features were selected using the *F*-score and SFFS algorithms. With an accuracy of 80% in distinguishing between tinnitus patients and healthy subjects, our results show that thirteen brain regions can effectively be used to differentiate patients with tinnitus from healthy subjects. These regions include the bilateral hypothalamus, right insula, bilateral STG, left rostral middle frontal gyrus, bilateral ITG, right IPL, right transverse temporal gyrus, right MTG, right cingulate gyrus, and left SFG. The AUC determined by ROC curve analysis also indicates the superior performance of the hybrid feature selection algorithm combining the *F*-score, SFFS, and SVM methods.

### 4.1. Model Selection

Strategies for feature subset selection can be divided into three categories: the exhaustion, heuristic, and random strategies [29]. In theory, the optimal feature subset can be found only using the exhaustion strategy. For small-scale feature subsets, the exhaustion method is one of the best choices for optimal feature selection. However, with increasing feature number, the computational complexity of the exhaustion method increases exponentially. Thus, for relatively high-dimensional data, as in this study, the exhaustion strategy cannot feasibly be applied. The random strategy includes a genetic algorithm, a simulated annealing algorithm, and a beam search algorithm [30]. It is suitable for studies with a flexible number of features. However, this strategy could not be used in the present study since the number of features was predefined according to previous reports.

The heuristic strategy was applied in this study. This strategy combines the advantages of the former two strategies. It is characterized by high accuracy and efficiency in feature subset searching. This strategy supports forward, backward, and combined search methods according to the direction of the search. Typically, the sequential forward search (SFS), SFFS, and sequential backward floating search (SBFS) strategies are commonly used [26, 31]. SFS is a bottom-up search strategy. During the feature subset search procedure, it adds the top feature to the selected feature subset until it meets the defined criteria. However, features that have been added cannot be excluded in the SFS strategy, which leads to a local maximum and may not be conducive to the extraction of an optimal feature set. SFFS and SBFS are flexible strategies for feature selection (i.e., features may be included and excluded flexibly) that avoid the generation of local maxima to a certain extent [32–34]. The purpose of this study was to select a limited number of brain regions among many that have been previously reported to effectively differentiate tinnitus patients from healthy subjects. Thus, it was of importance to first add brain regions with the most effectiveness in the selection model and then modify the features flexibly. Considering the *F*-scores calculated prior to the feature subset search procedure, the SFFS strategy was more suitable. Thus, the bottom-up SFFS strategy was applied. Based on the superior classification performance and good generalization performance of the SVM classifier, the SVM method was further applied in this study.

In this study, 5-fold cross-validation and a grid search were applied to train data during the calculations for optimal parameter (*C*, *γ*) selection. The search range of *C* and *γ* was defined as log_2_*C* = {−5, −4, ⋯, 4, 5} and log_2_*γ* = {−5, −4, ⋯, 4, 5}, respectively. Due to the limited number of features and enrolled subjects, i.e., 61 features and 102 subjects, a more detailed search range for optimal parameter definition and increased *K*-fold number may not generate better feature combinations. This hypothesis was further supported by our results. The optimal parameters (*C*, *γ*) and feature combinations with the highest average classification accuracy were detected. In this circumstance, combinations with more features should be discarded to limit the number of features. Thus, the combination of thirteen brain regions could be regarded as a superior result in this study.

### 4.2. Regions of Altered Brain Volume in Patients with Tinnitus

The pathophysiology of tinnitus is not limited to auditory brain regions but also includes nonauditory cortical and subcortical brain areas. Previous studies have reported various brain morphological alterations in patients with tinnitus. However, due to the inconsistency of those reported brain regions, it was difficult to generalize features of alteration in tinnitus patients. In this study, for the first time, we demonstrate a characteristic pattern of brain volume alteration using the SVM classifier. On the basis of sixty-one previously reported brain regions, 13 regions with the highest accuracy in classifying patients and healthy subjects in this study were selected and may indicate generalized features of alteration in tinnitus patients. This approach revealed the most likely cortical/subcortical morphological neuroimaging biomarkers characterizing tinnitus.

Among the brain regions listed in Table 4, both the right and left STG are listed as critical for SVM prediction. The anatomical proximity of these regions indicates that the brain volume of the STG may serve as a neuroanatomical biomarker in differentiating patients with tinnitus from healthy subjects. Our results are also in line with those reported by Meyer et al., who examined a large and homogeneous sample of tinnitus patients [4]. This group also found that a decreased cortical volume in the left STG was closely related to tinnitus distress. However, we should note that the left STG labeled in this study was not situated in the typical region of the primary auditory cortex. We also did not detect any anatomical changes in the primary auditory cortex, defined as the bilateral transverse temporal gyrus, or Heschl's gyrus, by the atlas of Desikan et al. [35]. Therefore, STG is a sensitive region but may not be the most important region [4]. However, studies of functional brain activity have demonstrated functional alterations in the STG and MTG in both chronic tinnitus and pulsatile tinnitus patients [36, 37]. As these regions are part of the self-perception network, which is also connected with the salience network, such anatomical alterations may also be part of a plastic effect associated with the functions of self-perception and awareness of tinnitus [38].

The MTG has also generally been reported in previous studies. Although the MTG is listed as one of the cortical morphological neuroimaging biomarkers characterizing tinnitus in this study, it did not have a high *F*-score for differentiating tinnitus patients from healthy controls. Boyen suggested that the GM volume of the MTG is increased in tinnitus patients with hearing impairment [5]. Since tinnitus is a very heterogeneous condition with respect to hyperacusis and the hearing loss status, we paid special attention to the clinical symptoms of the patients enrolled in this study. Tinnitus patients who applied for training and testing all had a normal hearing threshold without hyperacusis. Thus, this consideration may be the reason that the MTG was not selected earlier as one of the biomarkers in this study. Other brain areas that may be associated with hearing loss in the tinnitus groups, including the ventromedial prefrontal cortex (vmPFC) and cerebellum [9, 21], were also not identified in our study. Thus, our study also supports the idea that it is necessary to investigate tinnitus patients according to their clinical characteristics to minimize possible confounding factors induced by heterogeneous clinical conditions.

Anatomical and functional alterations in the limbic network in regions including the insula, parahippocampal gyrus, thalamus, amygdala, hippocampus, and cingulate gyrus [14, 39, 40] have commonly been reported in previous studies. This network may not be directly associated with the generation of the tinnitus sound; however, it is closely related to negative emotional reactions to tinnitus (i.e., tinnitus-related distress) [11]. Additionally, the limbic network is responsible for the signal processing of tinnitus based on the “noise cancellation” mechanism. When the limbic network is compromised, tinnitus can be perceived by patients. Thus, morphological changes in the limbic network are considered critical indicators of tinnitus. As reported by Professor Leaver et al. [21], the morphology of the anterior insula is more closely related to tinnitus distress rather than tinnitus sound perception, anxiety, or depression. The parahippocampal gyrus and amygdala appear to be more responsive to sound in severe tinnitus patients than in mild-to-moderate tinnitus patients [41]. Additionally, according to the tinnitus model proposed by Husain et al., the insula is much more likely to be affected in tinnitus patients than the parahippocampal gyrus or amygdala, especially in cases of mild or habituated tinnitus [42]. This idea is further supported by our study. Since the average THI score of tinnitus patients in our study was 48.8, patients with severe, bothersome tinnitus did not account for the majority of our research group. Pearson's correlation analyses also revealed that the THI score was positively correlated with the volume of the right insula in moderate tinnitus. Thus, this may be the reason the insula was found as one of the most likely anatomical biomarkers in our group of tinnitus patients. However, the THI score cannot effectively measure the psychiatric state of tinnitus patients. We did not measure the psychological distress of the tinnitus patients. Additional studies are needed to further analyze the degree of distress in such patients and discuss the function of the limbic system.

Previous studies have mainly focused on measuring the cortical volume in the brain. However, subcortical structural changes, such as changes in the hypothalamus, have also been detected. In our study, the bilateral hypothalamus was identified as a critical structure in SVM prediction (Table 4). The hypothalamus is also part of the limbic system. Boyen et al. [5] found both decreased brain volume and decreased concentration in the bilateral hypothalamus in tinnitus patients with hearing impairment. However, few previous studies have reported anatomical changes in the hypothalamus. The meaning of the plastic effect on the bilateral hypothalamus is still unclear. Its clinical relevance needs to be investigated in future research.

We also recognize several limitations in this study. First, only brain region volumes were included as features in this study. However, the cortical/subcortical volume can also be revealed by two distinct neuroanatomical traits: thickness and surface area [4]. Achieving better results may rely on the use of distinct kinds of features; yet, in most previous studies, only volumetric changes were identified in tinnitus patients. As a result, we could apply only volume as a morphological feature due to the limited thickness and surface area data. Second, the datasets used for training and testing were relatively small. Abundant data diminish the risk of overfitting during the calculations. Due to the strict criteria applied for inclusion and exclusion, the amount of data in this study met the minimum standard for training. However, more robust results could be obtained with the enrollment of more subjects. Much more validated evidence of neuroimaging biomarkers for tinnitus patients might be extracted in future studies if more detailed features are included and calculations are based on larger datasets. Additionally, there was no measure of psychological distress or any psychiatric diagnosis for the tinnitus patients or healthy controls. The evaluation of distress is essential for analyzing the mechanism of brain structure alteration, especially in the limbic system. Additionally, further studies that specifically focus on the effect of aging in elderly tinnitus patients may be necessary.

## 5. Conclusions

By applying the machine learning SVM classification algorithm, we were able to differentiate tinnitus patients from healthy subjects. In more detail, our study provides a new and valuable method for the study of brain morphology in tinnitus—a hybrid feature selection algorithm combining the *F*-score and SFFS methods. Based on the SVM classification results, 13 cortical/subcortical brain regions that could effectively differentiate patients with tinnitus from healthy subjects were obtained. Although this method needs to be improved before it is applied in the clinic, these brain regions can serve as morphological neuroimaging biomarkers for patients with tinnitus. These findings contribute to the understanding of neuroanatomical alterations in tinnitus.

## Figures and Tables

**Table 1:** Characteristics of the participants.

	TP (*n* = 46)	HC (*n* = 56)	*p* value
Age (years)	22-63 (45.9 ± 11.9)	23-64 (41.6 ± 10.9)	0.059^a^
Gender (male/female)	20/26	19/37	0.413^b^
Tinnitus duration (months)	4-192 (62.5 ± 72.5)		
THI score	0-98 (48.8 ± 27.8)		

Data are presented as the ranges of min-max (means ± standard deviations). TP: tinnitus patients; HC: healthy controls. ^a^Two-sample two-tailed *t*-test. ^b^Chi-square test.

**Table 2:** Volumetric structures used in SVM classification model.

Brain region	Peak MNI (*x*, *y*, *z*)	References
Right hemisphere		
Ventromedial prefrontal cortex	2, 21, -15	Leaver et al. [21]
Dorsomedial prefrontal cortex	2, 38, 39	
Superior temporal gyrus	52, -41, 13	Boyen et al. [5]
	51, -4, -2	Aldhafeeri et al. [20]
	46, -15, -6	Schecklmann et al. [8]
	51, -6, -9	
	59, -1, -4	Meyer et al. [4]
Cingulate gyrus	4, 49, -5	Aldhafeeri et al. [20]
	10, 30, 22	
	5, -55, 28	
Middle temporal gyrus	48, -58, 6 63, 5, -17	Aldhafeeri et al. [20]
	49, -70, 13	Boyen et al. [5]
	46, -15, -6 51, -6, -9	Schecklmann et al. [8]
Parahippocampal gyrus	14, 5, -17	Aldhafeeri et al. [20]
	37, -35, -15	Meyer et al. [4]
Inferior temporal gyrus	47, -32, -17	Aldhafeeri et al. [20]
	55, -23, -24	Meyer et al. [4]
Rostral middle frontal gyrus	37, 51, 9	Meyer et al. [4]
	16, 21, -17	
Inferior parietal lobule	44, -63, 39	Meyer et al. [4]
	43, -66, 25	
	46, -56, 44	
Insula	36, 3, 1	Meyer et al. [4]
	39, 1, 1	
Superior temporal gyrus (primary auditory cortex, BA41)	43, -30, 10	Aldhafeeri et al. [20]
Cuneus	5, -77, 16	Meyer et al. [4]
Transverse temporal gyrus	43, -24, 3	
Pars orbitalis	45, -55, 43	
Supramarginal gyrus	59, -40, 24	Leaver et al. [21]
	57, -57, 27	Boyen et al. [5]
Occipital lobe	1, -84, -3	
Hypothalamus	5, -5, -11	
Superior frontal gyrus	11, 18, 59	Aldhafeeri et al. [20]
Middle frontal gyrus	48, 35, 20	
Inferior frontal gyrus	50, 19, -12	
Left hemisphere		
Superior temporal gyrus	-46, -34, 10	Boyen et al. [5]
	-63, -6, 1	Aldhafeeri et al. [20]
	-44, -12, -11	Schecklmann et al. [8]
	-58, -16, 6	
	-48, 9, -25	Meyer et al. [4]
	-47, 8, -26	
Cingulate gyrus	-14, 23, -13	Aldhafeeri et al. [20]
	-20, 5, 43	
	-4, -43, 29	
Middle temporal gyrus	-44, -12, -11	Schecklmann et al. [8]
Parahippocampal gyrus	-20, 2, -23	Aldhafeeri et al. [20]
Inferior temporal gyrus	-62, -12, -26	Aldhafeeri et al. [20]
	-44, -50, -12	Meyer et al. [4]
	-45, -50, -12	
Rostral middle frontal gyrus	-20, 56, -2	Meyer et al. [4]
	-23, 54, 16	
	-20, 55, -3	
Superior frontal gyrus	-7, 27, 55	Meyer et al. [4]
	-7, 52, 36	
	-11, 63, 19	Boyen et al. [5]
	-12, 65, 6	Aldhafeeri et al. [20]
Superior temporal gyrus (primary auditory cortex, BA41)	-42, -23, 10	Aldhafeeri et al. [20]
Transverse temporal gyrus	-51, -21, 4	Meyer et al. [4]
Hypothalamus	-4, -10, -6	Boyen et al. [5]
Middle frontal gyrus	-36, 35, 28	Aldhafeeri et al. [20]
Inferior frontal gyrus	-10, 63, 8	
Postcentral gyrus	-32, -31, 61	Meyer et al. [4]

MNI: Montreal Neurological Institute.

**Table 3:** The computation results with the highest classification accuracy from hybrid feature selection algorithms.

Dataset	# of original features	# of selected features	Accuracy (%)	
			Training set	Test set
TP+HC	61	13	80.49	80.00

TP: tinnitus patients; HC: healthy controls.

**Table 4:** SVM-derived brain regions that are critical to SVM prediction, ranked by their importance in SVM model.

Brain region	Peak MNI (*x*, *y*, *z*)	Volume in patients (mm^3^)	Volume in HC (mm^3^)
R hypothalamus	5, -5, -11	349.0 ± 45.5	383.5 ± 56.1
L hypothalamus	-4, -10, -6	89.4 ± 7.4	95.9 ± 10.0
R insula	36, 3, 1	306.9 ± 32.5	328.0 ± 39.9
R superior temporal gyrus	52, -41, 13	414.2 ± 65.3	438.8 ± 69.8
L rostral middle frontal gyrus	-20, 56, -2	372.7 ± 99.23	372.7 ± 99.23
R inferior temporal gyrus	55, -23, -24	313.4 ± 51.2	330.8 ± 49.0
R inferior parietal lobule	43, -66, 25	503.8 ± 77.0	469.9 ± 54.5
L superior temporal gyrus	-47, 8, -26	317.7 ± 33.4	310.4 ± 39.2
L inferior temporal gyrus	-62, -12, -26	207.0 ± 28.2	214.2 ± 22.2
R transverse	43, -24, 3	382.8 ± 43.3	400.5 ± 48.7
R middle temporal gyrus	49, -70, 13	280.7 ± 28.5	267.4 ± 50.1
R cingulate	10, 30, 22	370.0 ± 61.8	392.7 ± 66.6
L superior frontal gyrus	-11, 63, 19	347.4 ± 66.8	364.0 ± 67.5

## Data Availability

The MRI data used to support the findings of this study are available from the corresponding authors upon request.
